# Genetic and enzymatic characterization of two novel *bla*_NDM-36, -37_ variants in *Escherichia coli* strains

**DOI:** 10.1007/s10096-023-04576-y

**Published:** 2023-02-22

**Authors:** Wanshan Ma, Bo Zhu, Wen Wang, Qian Wang, Xiaodi Cui, Yujiao Wang, Xiutao Dong, Xiaofeng Li, Jianping Ma, Fang Cheng, Xiaohong Shi, Liang Chen, Siqiang Niu, Mingju Hao

**Affiliations:** 1 Department of Clinical Laboratory Medicine, The First Affiliated Hospital of Shandong First Medical University & Shandong Provincial Qianfoshan Hospital, Shandong Medicine and Health Key Laboratory of Laboratory Medicine, No. 16766 Jingshi Road, Lixia District, Jinan, China; 2 Xiamen Key Laboratory of Genetic Testing, Department of Laboratory Medicine, The First Affiliated Hospital of Xiamen University, Xiamen, China; 3 Department of Laboratory Medicine, The First Affiliated Hospital of Chongqing Medical University, No. 1 Friendship Road, Yuzhong District, Chongqing, China; 4 Department of Laboratory Medicine, Shandong Provincial Hospital Affiliated to Shandong First Medical University & Shandong Academy of Medical Sciences, Jinan, Shandong China; 5 School of Clinical Medicine, Jining Medical University, Jining, China; 6 Center for Discovery and Innovation, Hackensack Meridian Health, Nutley, NJ USA; 7 Department of Medical Sciences, Hackensack Meridian School of Medicine, Nutley, NJ USA

**Keywords:** Carbapenems, *E. coli*, *Bla*_NDM-36_, *Bla*_NDM-37_, Urinary tract infection

## Abstract

The widespread of different NDM variants in clinical Enterobacterales isolates poses a serious public health concern, which requires continuous monitoring. In this study, three *E. coli* strains carrying two novel *bla*_NDM_ variants of *bla*_NDM-36, -37_ were identified from a patient with refractory urinary tract infection (UTI) in China. We conducted antimicrobial susceptibility testing (AST), enzyme kinetics analysis, conjugation experiment, whole-genome sequencing (WGS), and bioinformatics analysis to characterize the *bla*_NDM-36, -37_ enzymes and their carrying strains. The *bla*_NDM-36, -37_ harboring *E. coli* isolates belonged to ST227, O9:H10 serotype and exhibited intermediate or resistance to all β-lactams tested except aztreonam and aztreonam/avibactam. The genes of *bla*_NDM-36, -37_ were located on a conjugative IncHI2-type plasmid. NDM-37 differed from NDM-5 by a single amino acid substitution (His261Tyr). NDM-36 differed from NDM-37 by an additional missense mutation (Ala233Val). NDM-36 had increased hydrolytic activity toward ampicillin and cefotaxime relative to NDM-37 and NDM-5, while NDM-37 and NDM-36 had lower catalytic activity toward imipenem but higher activity against meropenem in comparison to NDM-5. This is the first report of co-occurrence of two novel *bla*_NDM_ variants in *E. coli* isolated from the same patient. The work provides insights into the enzymatic function and demonstrates the ongoing evolution of NDM enzymes.

**Supplementary Information:**

The online version contains supplementary material available at 10.1007/s10096-023-04576-y.

## Introduction

The plasmid-encoded New Delhi metallo-β-lactamase (NDM) is one of the most common carbapenemases worldwide [1]. Its emergence heralds a new era of antibiotic resistance due to the ability to hydrolyze almost all known β-lactam antibiotics and the rapid worldwide dissemination [2]. In 2009, the first NDM variant (NDM-1) was reported in *Klebsiella pneumoniae* isolated from a Swedish patient of Indian origin who had a urinary tract infection [3]. Following the first report, 41 different NDM variants have been identified in numerous species of *Enterobacteriaceae* and common nonfermentative Gram-negative bacilli [4] (NDM-1 to NDM-41; NDM-32 is assigned but without any information in NCBI).

The continuous evolution of NDM enzymes under the selection pressure could foster the emergence of new variants that possess different catalytic activities toward β-lactam agents [5]. For example, the NDM-5 variant showed enhanced hydrolytic activity compared with NDM-1 [6], and circular dichroism spectroscopy data revealed significant changes in the secondary structure of NDM variants [7]. Thus, a close surveillance of NDM-producing pathogens should be considered for continuous monitoring of the spread of NDM variants. Here, we describe the first detection of two novel NDM enzymes, designated NDM-36 and NDM-37, recovered from a patient with refractory urinary tract infection in China during 2020.

## Methods

### Bacterial strains

Three carbapenem-resistant *E. coli* strains (*bla*_NDM_ positive) were isolated from the urine samples of a 62-year-old female patient with unilateral indwelling ureteral stents. The patient underwent cystectomy and chemotherapy for recurrence of ovarian and fallopian cancer three months ago. The first *E. coli* strain (JNQH497-NDM-37) was recovered in an outpatient clinic. Based on the antibiotic susceptibility testing results, empirical levofloxacin treatment (500 mg qd) was then started for urinary tract infection. After 3 weeks, the second strain (JNQH498-NDM-36) was isolated from the urine during hospital admission. The patient was then given meropenem (1000 mg intravenously [i.v.] q8h). The third *E. coli* strain (JNQH462-NDM-36) was identified from the urine 5 weeks after admission. Her conditions were improved after the removal of ureteral stents via cystoscopy, continuous meropenem treatment as well as implementation of nutritional support. The patient was discharged home on hospital day 16. Ethics committee approval of this study was obtained from the institutional review board of the First Affiliated Hospital of Xiamen University, and informed consent from the patient was also obtained.

### Antimicrobial susceptibility testing (AST)

MICs for all the tested strains were determined by broth microdilution method using a bacterial inoculum of 5 × 10^5^ CFU/ml according to CLSI performance standards. For ceftazidime-avibactam (CAZ-AVI) and aztreonam-avibactam (ATM-AVI) MICs evaluation, AVI was tested at a fixed concentration of 4 mg/L, while CAZ and ATM were added at different concentrations ranged from 0.0312 to 64 mg/L and 0.0156 to 32 mg/L, respectively.

### Cloning of *bla*_NDM_ variants

The promoter and full length of the *bla*_NDM_ genes were amplified with primers NDM-F-*EcoRI* (5′-CCGGAATTCTTGAAACTGTCGCACCTCAT-3′) and NDM-R-*XbaI* (5′-CTAGTCTAGAACGCCTCTGTCACATCGAA-3′) using PrimSTAR Max DNA Polymerase (Takara, China). After restriction enzyme digestion, the PCR products were ligated to the vector PET28a to generate PET28a-NDM-5, PET28a-NDM-36, PET28a-NDM-37 respectively. The correct constructs were confirmed by Sanger sequencing, followed by transformation into *E. coli* DH5α. Antimicrobial susceptibilities of these constructs were determined as described above. The empty pET28a plasmid was used as a control.

### Expression of the NDM proteins

The sequences of NDM-5, -36, -37 without peptide signal region were amplified by PCR using primers *EcoRI*-NDM (29-271AA)-F (5′—CCGGAATTCATGGAATTGCCCAATAT—3′) and *HindIII*-NDM (29-271AA)-R (5′—CCCAAGCTTTCAGCGCAGCTTGTCGGCC -3′), followed by insertion into plasmid pET28a in *E. coli* BL21 (Invitrogen). Protein was expressed in *E. coli* strain BL21 grown in LB at 37 ℃. Once an OD600 of 0.4–0.6 was reached, 0.1 mM ZnCl_2_ and 0.5 mM isopropyl-β-d-thiogalactoside were added into the LB medium. The temperature was then lowered to 20 ℃, and the expression was allowed to occur overnight. Later, the cells were lysed by sonication, and the supernatant was loaded to a HisTrap™ HP column (GE Healthcare, Little Chalfont, UK). Finally, the purified proteins were dialysised in the buffer (50 mM HEPES, 100 μM ZnCl_2_, 250 mM NaCl, and 20 mg/L BSA) at 4 °C overnight [8].

### Steady-state kinetic parameters

Steady-state kinetic experiments were performed following the hydrolysis of the β-lactams at 25 °C in 50 mM HEPES (pH 7.5) plus 100 μM ZnCl_2_. The data of the real-time absorbances of meropenem (298 nm), imipenem (297 nm), ceftazidime (257 nm), aztreonam (318 nm), cefotaxime (264 nm), cefepime (254 nm), piperacillin (232 nm), ceftriaxone (240 nm), and ampicillin (235 nm) were collected with a SHIMADZU UV2550 spectrophotometer (Kyoto, Japan). Kinetic parameters were determined under initial-rate conditions using the GraphPad Prism 8.1 software to generate Michaelis–Menten curves or by analyzing the complete hydrolysis time courses [9].

### Conjugation experiment

Conjugation experiments were performed using *E. coli* J53AziR as recipients as previously described [10]. Briefly, overnight cultures of the donor strain (JNQH497, 498) and the recipient strains were mixed (1:1) and applied to 0.45-μm filter paper respectively, which were then placed on an LB agar plate, followed by overnight culture at 37 ℃. Transconjugants were selected on Mueller–Hinton agar containing sodium azide (100 mg/L)/meropenem (2 mg/L) for transconjugates. The selected transconjugants were confirmed by PCR targeting the *bla*_NDM_ gene. Conjugation frequency was calculated by dividing the number of transconjugants by the number of recipient cells.

### Pulsed-field gel electrophoresis (PFGE) 

To further explore the relatedness of JNQH497, 498, 462 strains, we used PFGE to analyze the genetic relatedness. PFGE of Xbal-digested genomic DNA samples were performed with a CHEF MAPPER XA apparatus (Bio-Rad, USA), as previously described [11].

### Whole-genome sequencing (WGS)

The strains were subject to next generation sequencing using the Illumina HiSeq system (Illumina, San Diego, CA, USA). Genomic DNA was isolated using a WizardR Genomic DNA Purification Kit (Promega, Madison, WI, USA). Sequencing reads were de novo assembled using Spades 3.12.0 [12]. To resolve the complete plasmid sequence carrying *bla*_NDM_ in JNQH497 and JNQH498, the Oxford Nanopore (MinION system) sequencing was conducted and assembled with Illumina sequences to achieve a high-quality genome assembly. The hybrid assembly was performed using Unicycler v0·5.0 [13]. The whole-genome sequences were annotated by Prokka [14] automatically followed by manual curation.

### Genomic analysis

In silico multi-locus sequence typing was performed using MLST 2.0 [15], while the acquired antimicrobial resistance genes were identified using the ABRicate program (https://github.com/tseemann/abricate) to query the CARD database (http://genomicepidemiology.org/). Identification of serotypes was performed using ECTyper (v.1.0) [16] with default parameters (https://github.com/phac-nml/ecoli_serotyping). The plasmid replicons in the sequenced isolates were identified using PlasmidFinder 2.0 [17]. Single-nucleotide polymorphisms (SNPs) and small insertions (INS) were detected using Snippy v3.2 (https://github.com/tseemann/snippy) by mapping the Illumina sequence reads of the JNQH498 and JNQH462 to the complete chromosome sequence of isolate JNQH497. Blastn was used to examine sequences homologous to the sequenced plasmids in the NCBI database. Comparison between homologous plasmids was conducted using CGview server [18]. OriT Finder was used to determine the conjugation module [19]. In order to examine the plasmid replicon distribution of *bla*_NDM_-bearing plasmids, plasmid sequences were downloaded from NCBI (https://ftp.ncbi.nlm.nih.gov/refseq/release/plasmid) and compared. Plasmid distance trees were generated using Mashtree [20]. The amino acid sequences of *bla*_NDM_ were retrieved from BLDB [4] and NCBI database, and were aligned using Clustal Omega [21]. Evolutionary analyses were conducted in MEGA X for inferring maximum-likelihood phylogenies [22].

## Results

### Antimicrobial susceptibility testing

Broth microdilution susceptibility testing showed that JNQH497, JNQH462, and JNQH498 were resistant to ampicillin, cefazolin, cefotaxime, ceftazidime, meropenem, amikacin, levofloxacin, and ceftazidime/avibactam, but were susceptible to aztreonam (MICs, ≤ 0.25 mg/L), aztreonam/avibactam (MICs, ≤ 0.008 mg/L), and colistin (MICs = 0.125 mg/L) (Table 1). NDM-36-producing isolates (JNQH498, JNQH462) exhibited decreased susceptibility to imipenem and meropenem comparing with the isolate harboring NDM-37 (JNQH497). Of note, JNQH498 and JNQH462 were resistant to imipenem (MIC, 8 mg/L), while JNQH497 was intermediate to imipenem with an MIC value of 2 mg/L.

**Table 1:** Antibiotic susceptibility profiles of NDM-carrying clinical isolates and *E. coli* DH5α transformants

Strains	MICs (mg/L)												
	AMP	CZO	CTX	CAZ	FEP	ATM	IMP	MEM	CAZ/AVI	ATM/AVI	LEV	AMK	COL
Wide-type strains													
JNQH497(NDM-37)	1024	256	64	≥ 1024	8	≤ 0.25	2	4	≥ 128	≤ 0.008	2	64	0.125
JNQH498(NDM-36)	≥ 2048	≥ 1024	512	≥ 1024	64	≤ 0.25	8	8	≥ 128	≤ 0.008	16	64	0.125
JNQH462(NDM-36)	≥ 2048	≥ 1024	> 512	≥ 1024	128	≤ 0.25	8	8	≥ 128	≤ 0.008	16	64	0.125
J53	8	2	≤ 0.125	≤ 0.125	≤ 0.125	≤ 0.25	≤ 0.06	≤ 0.06	0.25	≤ 0.008	≤ 0.03	2	0.125
DH5α	2	2	≤ 0.125	≤ 0.125	≤ 0.125	≤ 0.25	≤ 0.06	≤ 0.06	0.25	≤ 0.008	≤ 0.03	2	0.125
E. coli J53 transconjugants													
JNQH497(NDM-37)	≥ 2048	≥ 1024	64	≥ 1024	32	≤ 0.25	8	32	≥ 128	≤ 0.008	0.25	2	0.125
JNQH498(NDM-36)	≥ 2048	≥ 1024	128	≥ 1024	64	≤ 0.25	8	16	≥ 128	≤ 0.008	0.25	2	0.125
Cloning of blaNDM variants													
DH5α (pET-28a)	4	2	≤ 0.125	0.5	≤ 0.125	≤ 0.25	≤ 0.06	≤ 0.06	0.25	0.03	≤ 0.03	4	0.125
DH5α (pET-28a-NDM-5)	≥ 2048	≥ 1024	128	≥ 1024	128	≤ 0.25	16	32	≥ 128	≤ 0.008	0.125	4	0.125
DH5α (pET-28a-NDM-37)	≥ 2048	≥ 1024	64	≥ 1024	32	≤ 0.25	8	32	≥ 128	≤ 0.008	0.25	2	0.125
DH5α (pET-28a-NDM-36)	≥ 2048	≥ 1024	128	≥ 1024	64	≤ 0.25	8	16	≥ 128	≤ 0.008	0.25	2	0.125

*AMP* ampicillin, *CZO* cefazolin, *CTX* cefotaxime, *CAZ* ceftazidime, *FEP* cefepime, *ATM* aztreonam, *IPM* imipenem, *MEM* meropenem, *AVI* avibactam, *LEV* levofloxacin, *AMK* amikacin, *COL* colistin

### Identification of *bla*_NDM-36, -37_ in *E. coli* strains

Whole-genome sequencing analysis showed all strains belonged to sequence type 227 (ST227) and O9:H10 serotype. Two novel NDM-encoding genes were identified in JNQH462 and JNQH498, designated *bla*_NDM-36_ (NG_076641.1) and *bla*_NDM-37_ (NG_076642.1), respectively. Relative to *bla*_NDM-5_, *bla*_NDM-37_ contained one missense point mutations at positions 781 (C → T), generating amino acid substitution His261Tyr. Relative to NDM-37, NDM-36 contained one additional missense point mutation at position 698 (C → T), resulting in amino acid substitution Ala233Val.

**Fig. 1:**
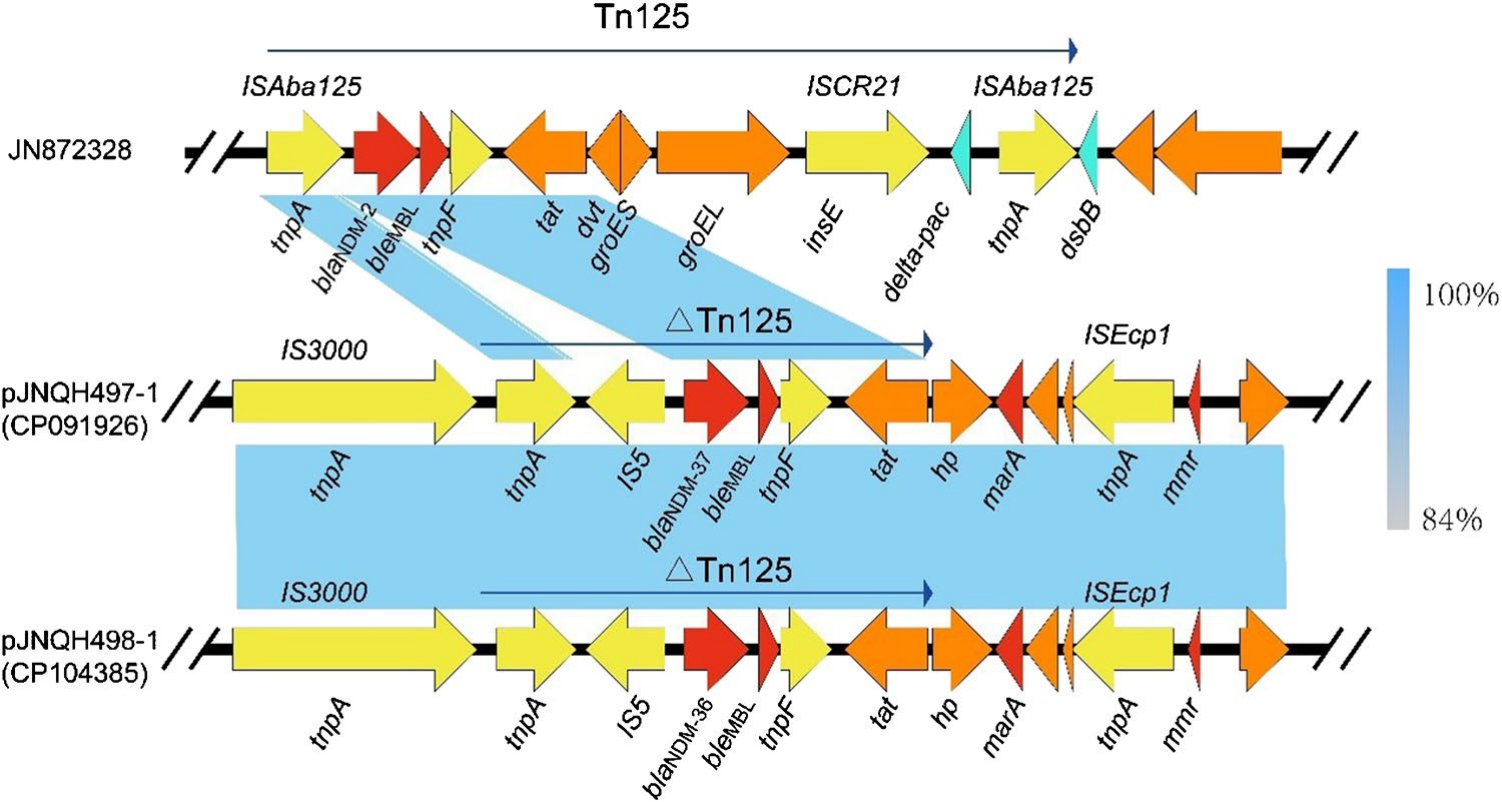
Linear genetic context of *bla*_NDM-37_ on IncHI2-type plasmid pJNQH497-1. The region of *bla*_NDM-2_ in Tn125 on JN872328 is shown for comparison. Genes, mobile elements, and other features are colored based on their functional classification. Light blue shading indicates shared regions of homology

**Fig. 2:**
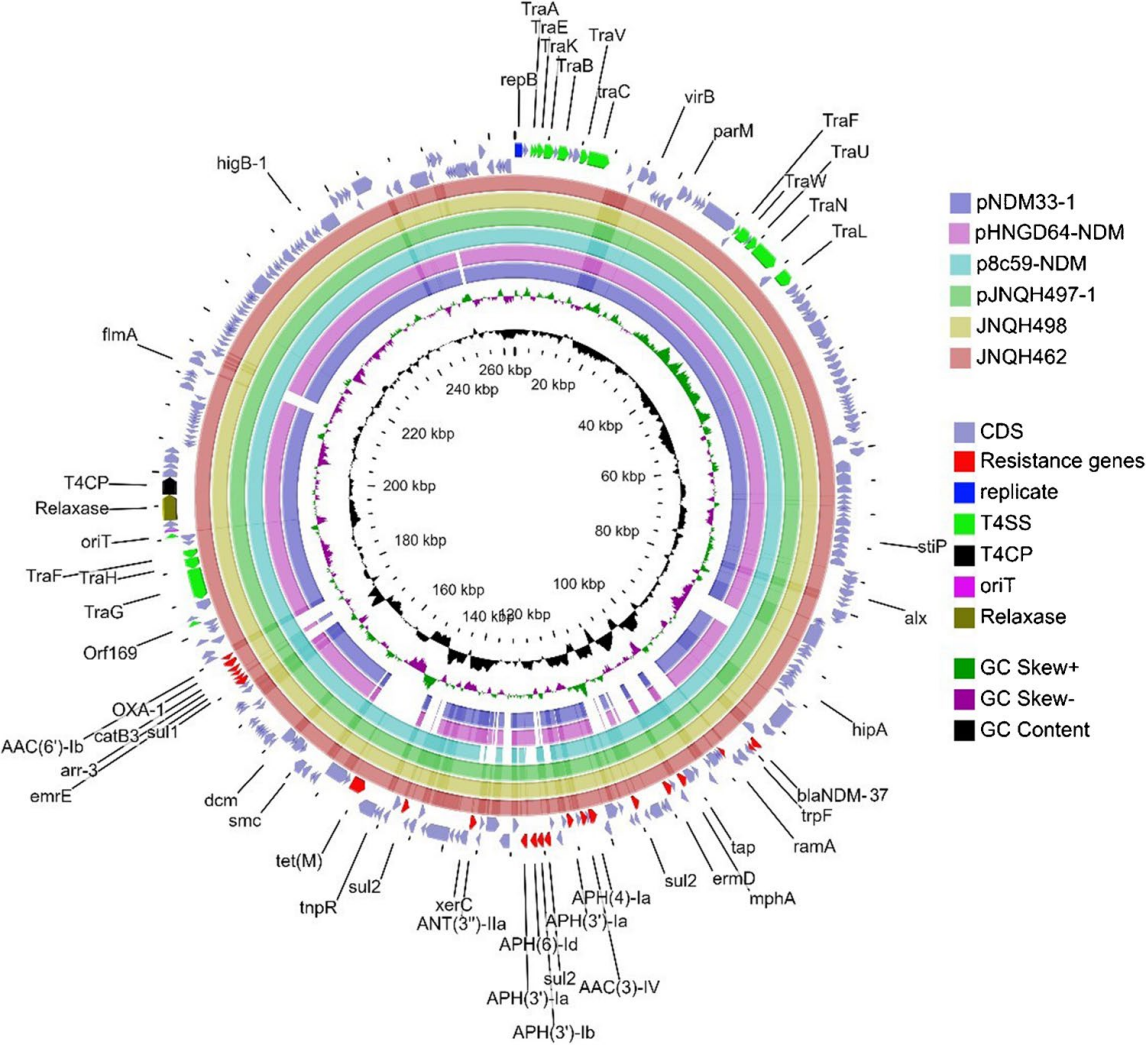
Comparison of pJNQH497-1 (CP091926), pJNQH498-1 (CP104385), p8c59NDM (NZ_MT407547.1), pHNGD64-NDM (NZ_MW296099.1), pNDM33-1 (NZ_CP076648.1), and draft genome sequences of JNQH462. Open reading frames (ORFs) of pJNQH497-1 are shown as the outermost ring, with plasmid replicons, plasmid transfer associated (oriT, T4SS, T4CP, relaxase), and antimicrobial resistance genes highlighted. pJNQH497-1 was used as the reference for Blastn comparison

Combination of long-read and short-read sequencing revealed that strain JNQH497 harbored a 4.75-Mb chromosome and two plasmids, designated pJNQH497-1 (263-Kb), and pJNQH497-2 (103.3-Kb). *bla*_NDM-37_ was carried by pJNQH497-1 which belongs to IncHI2-type plasmid. JNQH498 harbored a 4.75-Mb chromosome and one plasmid, designated pJNQH498-1 (263-Kb). *bla*_NDM-36_ was carried by pJNQH498-1 which also belongs to IncHI2-type plasmid. *bla*_NDM-36_ and *bla*_NDM-37_ were located in an ΔTn*125*-like region with the structure of “*tnpA-IS5-bla*_NDM-37_*-ble*_MBL_*-trpF-tat*,” containing Tn*3* family transposase IS*3000* upstream and IS*1380* family transposase ISEcp1 downstream (Fig. 1). In addition to the truncated Tn*125* cluster, multiple aminoglycoside resistance genes [*APH(4)-Ia, AAC(3)-IV, APH(3′)-Ia, APH(3′)-Ib, APH(6)-Id, ANT(3′)-IIa, AAC(6′)-Ib*], fluroquinolone [*rmpA, AAC(6′)-Ib-cr*), and sulphonamide resistance genes (*sul1, sul2*) were also found on the same plasmid (Fig. 2). Further, *OXA-1* and *TEM-150* genes producing ESBL were located on pJNQH497-1 and pJNQH497-2 respectively in JNQH497. *OXA-1* gene was located on pJNQH498-1 while TEM-150 was absent in JNQH498.

### Comparison of sequences and PFGE patterns

Comparison of complete sequences of JNQH497 and JNQH498 showed the *bla*_NDM-36_ harboring plasmid (pJNQH498-1) was almost the same as the *bla*_NDM-37_ harboring plasmid (pJNQH497-1, 99.99% nucleotide identity and 100% coverage) (Fig. 2). There were only 13 SNPs between the two plasmids including the c.698C > T in *bla*_NDM_ gene. Blastn analysis revealed that pJNQH497-1 was almost identical (100% query coverage and 99% identity) to the plasmid p8C59-NDM (NZ_MT407547.1) and displayed high similarity with pHNGD64-NDM [23] (NZ_MW296099.1, 89% query coverage and 99.8% identity) and pNDM33-1 [24] (NZ_CP076648.1, 86% query coverage and 99.9% identity). p8C59-NDM, pHNGD64-NDM, and pNDM33-1 also belonged to IncHI2-type and were carried by *E. coli* isolated from animal sources in China; however, they all harbored *bla*_NDM-5_, and the host *E. coli* strains belonged to ST10, ST4063, and ST48, respectively. In addition, a premature stop codon was introduced into the coding region of *ompD* (c.238G > T p.Glu80*) in JNQH498 and JNQH462 strains while it was absent in JNQH497 (Table S1).

**Fig. 3:**
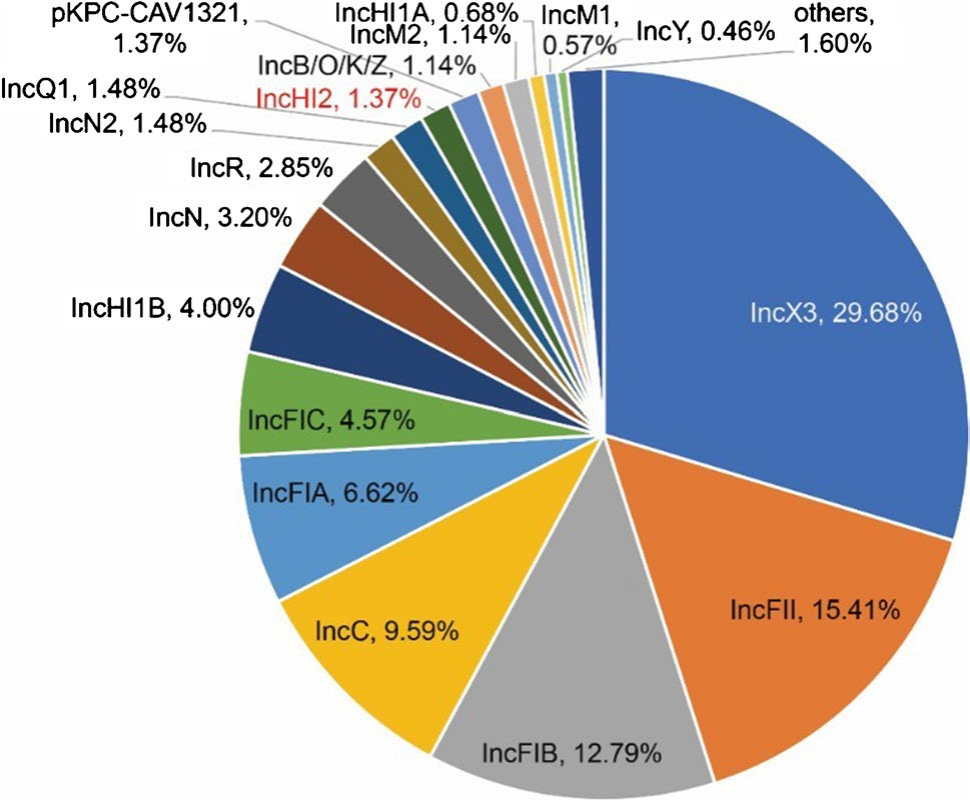
The plasmid replicon distribution of *bla*_NDM_-bearing plasmids from NCBI reference sequences

**Fig. 4:**
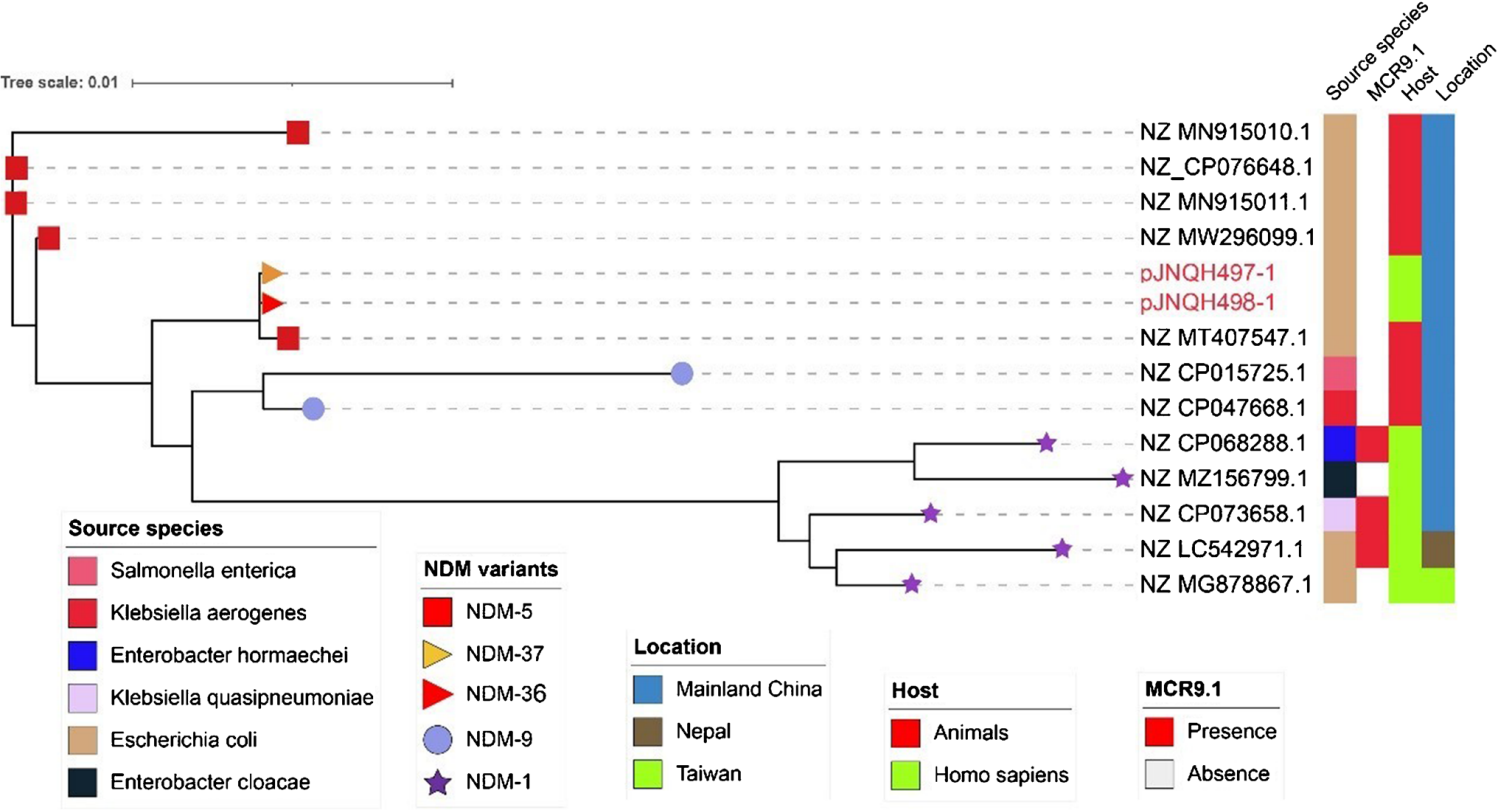
Phylogenetic analysis of 13 sequences of IncHI2-type plasmids harboring *bla*_NDM._ Colors in columns illustrated source species, presence of *mcr* resistance genes, host, and collection location

The major plasmid types carrying *bla*_NDM_ from reference NCBI database (*n* = 876) included IncX3 (29.68%), IncFII (15.41%), IncFIB (12.79%), and IncC (9.59%) (Fig. 3). Twelve *bla*_NDM_ harboring plasmids were found to be IncHI2 type (1.37%), which were mainly found in mainland China with the exception of two plasmids from Taiwan and Nepal respectively. Notably, three *bla*_NDM_ bearing IncHI2 plasmids also coharbored *mcr9.1* colistin resistance genes (Fig. 4).

**Fig. 5:**
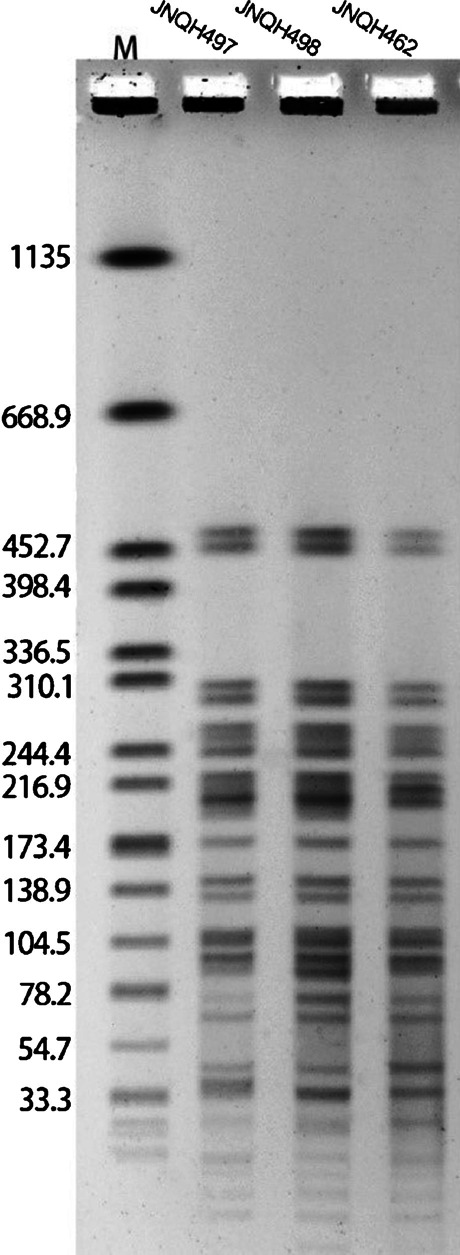
PFGE patterns of JNQH497, JNQH498, and JNQH462 strains digested with XbaI. Sizes of the bands of H9812 are represented on the left of lane M

The PFGE showed JNQH497, 498, 462 had highly similar band patterns which indicated they were closely related (Fig. 5).

### Transferability of *bla*_NDM_ harboring plasmids and conjugation module analysis

Conjugation assays showed the *bla*_NDM-36_ and *bla*_NDM-37_ harboring IncHI2-type plasmids were successfully transferred into *E. coli* J53 from JNQH498 and JNQH497 strains. *E. coli* J53 transconjugants acquired resistance to levofloxacin and most β-lactam antibiotics except aztreonam/avibactam (Table 1), which indicated the resistant markers to fluroquinolones were co-transferred with *bla*_NDM_ genes. The conjugation frequency was 10^−3^ per recipient cell for JNQH497, whereas it was only 10^−8^ for JNQH498. Conjugation module analysis revealed the conjugation genes are in two separate regions: transfer region 1 carries the origin of transfer site (oriT), type IV coupling protein gene (T4CP), and genes encoding the relaxase and some type IV secretion components. Region 2 encodes most type IV secretion proteins (Fig. 2). Blastn analysis revealed all the conjugation modules were also found in JNQH498, JNQH462 strains.

### Expression of the NDM proteins and enzyme activity analysis

Susceptibility testing of pET28a constructs showed that expression of the *bla*_NDM-36_ and *bla*_NDM-37_ genes in *E. coli* DH5α conferred resistance to most of the tested β-lactams except aztreonam and ATM/AVI. Kinetic data showed that NDM-36 had higher affinity to cefotaxime than that of NDM-37, with the Km value reduced by 82.62 μM, whereas NDM-36 displayed slightly lower affinity than those of NDM-5, -37 for imipenem and meropenem. The kcat/Km ratio for ampicillin and cefotaxime of NDM-36 was higher than those of NDM-37, -5, but imipenem kcat/Km ratio was slighter higher than those of NDM-37, -5. In comparison to NDM-5, although NDM-36, -37 had lower kcat/Km ratio for imipenem, they had higher kcat/Km ratio for meropenem. These results suggested NDM-36 had higher hydrolytic activity toward ampicillin and cefotaxime relative to NDM-37, -5, and that NDM-37, -36 had lower catalytic activity against imipenem but higher activity against meropenem relative to NDM-5 (Table 2).

**Table 2:** Steady-state kinetic parameters of purified NDM-5, NDM-36 and NDM-37 enzymes

β-Lactam	NDM-5			NDM-36			NDM-37		
	Km (μM)	kcat (s^−1^)	kcat/Km (μM^−1^ s^−1^)	Km (μM)	kcat (s^−1^)	kcat/Km (μM^−1^ s^−1^)	Km (μM)	kcat (s^−1^)	kcat/Km (μM^−1^ s^−1^)
Ampicillin	98.47	337.071	3.423	71.32	287.295	4.029	93.12	185.77	1.995
Cefotaxime	38.46	146.679	3.814	25.58	122.428	4.786	108.2	193.318	1.787
Ceftazidime	30.07	107.713	3.582	44.93	108.004	2.404	31.23	91.746	2.938
Cefepime	33.84	77.337	2.285	19.41	42.304	2.179	18.53	37.497	2.024
Aztreonam	ND	ND	ND	ND	ND	ND	ND	ND	ND
Imipenem	73.02	278.501	3.814	100	145.021	1.45	55.53	126.934	2.286
Meropenem	67.84	133.13	1.962	98.52	287.295	2.916	25.89	82.909	3.202

*ND* not detectable due to a low initial rate of hydrolysis

### Phylogenetic analysis of NDM protein sequences

**Fig. 6:**
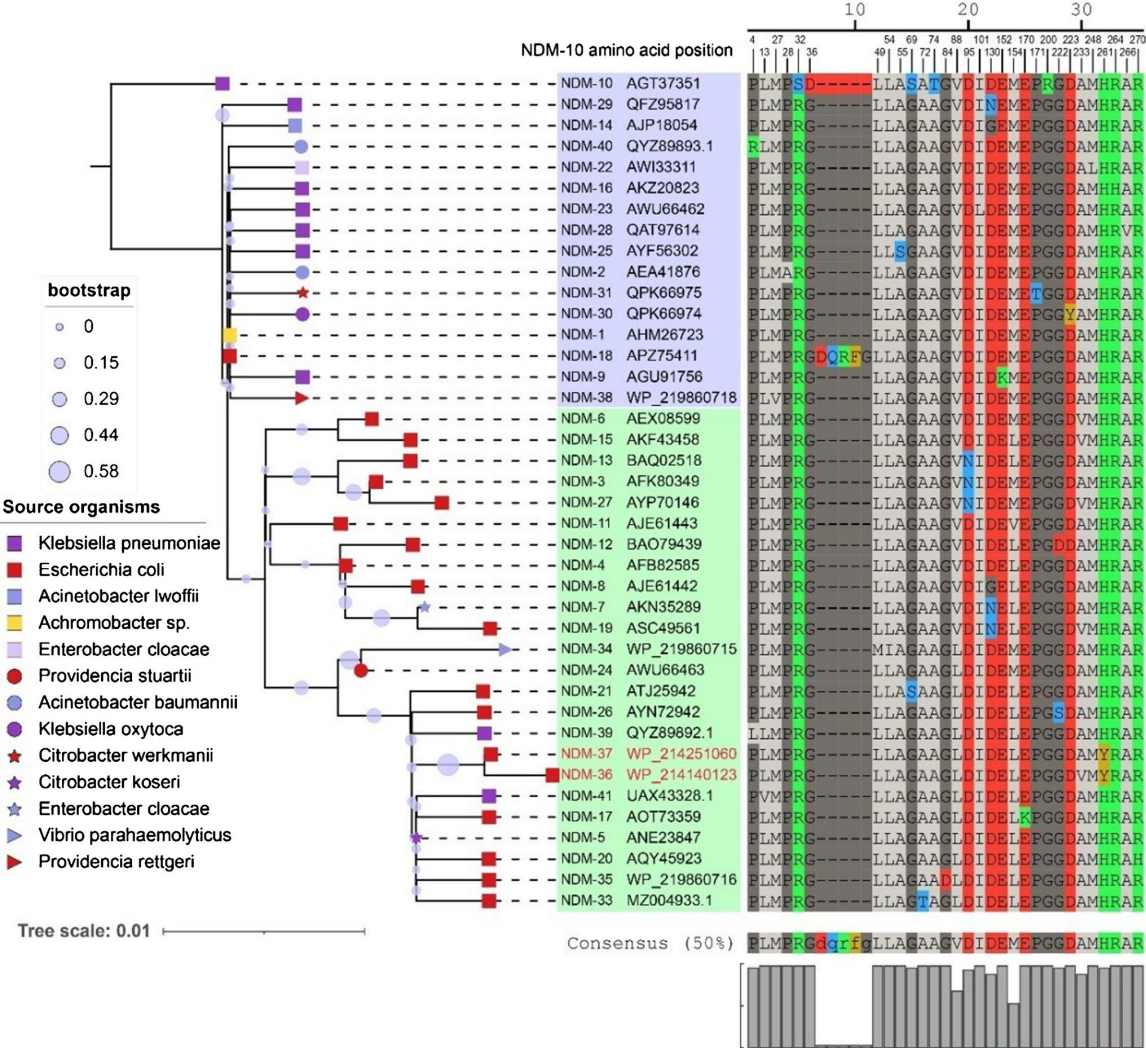
Evolutionary analysis and genetic variations among the NDM variants and its first source of spread. The percentage of replicate trees in which the associated taxa clustered together in the bootstrap test (1000 replicates) are shown next to the branches. Schematic representation of *bla*_NDM-1_ gene in the alignment showing the mutations at various nucleotide positions leading to the occurrence of NDM variants. Each unique color of NDM variants in the column showing mutant residues at different position

Phylogenetic analysis of the protein sequence of NDM variants is represented in Fig. 6. Evolutionary analysis of the amino acid sequences showed the amino acids were substituted at 30 polymorphic sites except for NDM-18, which has five amino acids tandem repeat (QRFGD) at positions 44 to 48 relative to NDM-1 [25]. The NDM variants had most hotspot mutation at amino acid positions 88, 154, and 130 (Fig. 6).

## Discussion

The widespread of NDM variants among *E. coli* strains and other *Enterobacteriaceae* isolates represents a large threat to the public health globally [26]. To date, a total of 41 NDM variants have been named and described, of which 40 sequences have been deposited in the GenBank database. In this study, two novel *bla*_NDM_ variants were carried by *E. coli* strains isolated from the same patient. Sequence analysis showed that NDM-37 differed from NDM-5 by a single amino acid substitution (His261Tyr) due to one missense point mutations at positions 781 (C → T). One additional missense point mutation at position 698 (C → T) in *bla*_NDM-37_ resulting in NDM-36. As such, this study provides a good explanation to increase our understanding that *bla*_NDM_ variants are undergoing continuous evolution and thus need to be closely monitored.

WGS revealed the new variants of *bla*_NDM-36, -37_ were located on a conjugational IncHI2-type plasmid. IncHI2 plasmids are larger than most of the other conjugative plasmids and have been found to be associated with various resistance genes including *mcr*, ESBL, and carbapenemase encoding genes in *Enterobacteriaceae* [27–29]. Complete transfer operons were identified in the plasmids, which is consistent with the finding that the *bla*_NDM-36_ and *bla*_NDM-37_ harboring IncHI2-type plasmids can be transferred by conjugation. In addition, the identification of highly similar IncHI2 plasmids in different *E. coli* STs suggested this plasmid had horizontal inter-species transfer between different *E. coli* clones, probably due to various antibiotic selection pressures as the plasmid contained multiple resistance genes.

It is noteworthy to mention, as shown in Table 1, most of the antibiotic susceptibility profiles of NDM-carrying clinical isolates were consistent with those of the corresponding *E. coli* DH5α transformants and J53 transconjugants, except for imipenem and meropenem. Compared with JNQH497, a premature stop codon was introduced into the coding region of *ompD* (c.238G > T p.Glu80*) in JNQH498 and JNQH462 strains. *OmpD* had been reported to be the main mechanism that mediated reduced susceptibility to imipenem in *Enterobacter spp* [30]. As such, it is speculated the premature stop codon likely accounts for the inconsistency of imipenem and meropenem susceptibility between pET28a-NDM-36 and pET28a-NDM-37 as compared to the corresponding source isolates. In addition, considering these isolates having MICs for meropenem ≤ 8 mg/L, the patient was given high-dose extended-infusion meropenem for urinary tract infection [31]. However, due to the excellent in vitro activity of ATM/AVI and the carriage of ESBL encoding genes, utility of aztreonam in combination with ceftazidime-avibactam might be one promising treatment strategy [32].

In summary, this study identified two novel NDM-type β-lactamases, NDM-36 and NDM-37, from *E. coli* strains isolated from a patient with refractory urinary tract infection. To the best of our knowledge, this is the first report describing two novel NDM variants detected from the same patient. This work extended our understanding of enzymatic function and demonstrated the ongoing evolution of NDM enzymes. Emergence of new NDM variants could be driven by de novo resistance evolution. A close surveillance of NDM-producing pathogens should be enacted for continued monitoring of the spread of NDM variants.

## Supplementary Information

Below is the link to the electronic supplementary material.

Supplementary file1 (DOCX 18 KB)

## Data Availability

The draft genome sequences of JNQH497, JNQH498, JNQH462 were deposited into NCBI Genome database under BioProject PRJNA702614. The complete genome sequences of JNQH497 and JNQH498 have been submitted to GenBank under the accession numbers of CP091925 ~ CP091927 and CP104384 ~ CP104385 respectively.
