# Supplementary material for: Genetic and enzymatic characterization of two novel blaNDM-36, -37 variants in Escherichia coli strains
Source: Eur J Clin Microbiol Infect Dis. 2023 Feb 22;42(4):471–80. doi: 10.1007/s10096-023-04576-y (PMC9998317; doi:10.1007/s10096-023-04576-y)
Supplement: Supplementary file 1 — Supplementary file1 (DOCX 18 KB) [file 10096_2023_4576_MOESM1_ESM.docx]

**Table S1. List of chromosomal SNPs and insertion differences among JNQH497, 498, 462 strains**

| Position in reference | JNQH497  (Reference) | JNQH498 | JNQH462 | TYPE | Effect with DNA (c.) and protein (p.) changes | Gene | Product |
| --- | --- | --- | --- | --- | --- | --- | --- |
| 128055 | T | G | G | SNP | synonymous_variant c.213T>G p.Pro71Pro | *yiaW_2* | Inner membrane protein YiaW |
| 343993 | T | C | C | SNP | missense_variant c.448T>C p.Cys150Arg | *ompR* | Transcriptional regulatory protein OmpR |
| 505934 | C | C | A | SNP | missense_variant c.293C>A p.Pro98His | *nanR_1* | HTH-type transcriptional repressor NanR |
| 944301 | T | C | C | SNP | missense_variant c.1004T>C p.Leu335Pro | *mltA* | Membrane-bound lytic murein transglycosylase A |
| 1106417 | G | T | T | SNP | missense_variant c.212G>T p.Ser71Ile | *ratA* | Ribosome association toxin RatA |
| 1204224 | T | G | G | SNP | missense_variant c.841T>G p.Ser281Ala | *hscA* | Chaperone protein HscA |
| 1385208 | C | C | T | SNP | - | *-* | - |
| 1681312 | T | C | C | SNP | missense_variant c.173T>C p.Ile58Thr | *xylB_2* | Xylulose kinase |
| 2050358 | T | T | G | SNP | missense_variant c.101A>C p.Glu34Ala | *dgcJ* | putative diguanylate cyclase DgcJ |
| 3134295 | T | T | TC | INS | frameshift_variant c.1126dupG p.Glu376fs | *dinG* | ATP-dependent DNA helicase DinG |
| 3171919 | G | T | T | SNP | stop_gained c.238G>T p.Glu80* | *ompD* | Outer membrane porin protein OmpD |
| 3473332 | C | T | C | SNP | missense_variant c.316G>A p.Asp106Asn | *dnaX* | DNA polymerase III subunit tau |

Nucleotide and amino acid changes in DNA and protein sequences of *E.coli* strains JNQH498 and JNQH462, with reference to the *E.coli* JNQH497 chromosome sequence; The symbol ’-’ in the column represents non-coding regions in the sequence.
